# Development of Tizanidine HCl-Meloxicam loaded mucoadhesive buccal films: *In-vitro* and *in-vivo* evaluation

**DOI:** 10.1371/journal.pone.0194410

**Published:** 2018-03-22

**Authors:** Muhammad Zaman, Muhammad Hanif, Zaib Ali Shaheryar

**Affiliations:** 1 Department of Pharmacy, Bahauddin Zakariya University, Multan, Pakistan; 2 Faculty of Pharmacy, The University of Lahore, Lahore, Pakistan; Brandeis University, UNITED STATES

## Abstract

The purpose of the study was to develop Tizanidine HCl (TZN) and Meloxicam (MLX) loaded bilayer mucoadhesive films intended for buccal administration, aiming to enhance the bioavailability. Bilayer films were prepared by solvent evaporation technique selecting arabinoxylan (ARX) as a sustained release (SR) layer forming polymer and hydroxypropyl methylcellulose (HPMC) E-15 as an immediate release (IR) layer-forming polymer. Prepared films were subjected to *in-vitro* drug release, surface morphology, mechanical strength, compatibility of the ingredients, drug contents, *ex-vivo* mucoadhesion strength and drug permeation. Crossover study design was applied to study the i*n-vivo* pharmacokinetics by using albino rabbits. Various pharmacokinetic parameters including AUC, C_max_, *t*_max_ and *t*_1/2_ of both drugs loaded in films were compared with standard solution/dispersion administered to the rabbits at the dose of 1mg/kg. The results unveiled instant release and permeation of MLX from IR layer, while good controlled release and permeation characteristics of TZN from SR films over 8 h. films were of uniform thickness with smooth surface and satisfactory mechanical strength. Mucoadhesion strength was sufficient to provide suitable contact time with mucosal membrane. The pharmacokinetic study exhibited prompt absorption of MLX with better AUC _0-t_ (6655.64 ng/ml*h vs 6538.99 ng/ml*h) and C_max_ (436.98 ng/ml vs 411.33 ng/ml) from oral dispersion. Similarly buccal films has shown enhanced half-life (9.91hr vs 2.51 hr), AUC _0-t_ (1043.4 ng/ml*h vs 149.1 ng/ml*h) and C_max_ (91.92 ng/ml vs 42.29 ng/ml) from oral solution. A statistical investigation disclosed a significantly improved pharmacokinetics of TZN and MLX after their absorption across buccal route following administration of buccal film *(p<0*.*05)*. ARX proved expedient and bilayer buccal films as a drug delivery system ascertained the dual effect of providing instant release of one active agent and persistent release of another one with improved pharmacokinetics.

## 1. Introduction

Buccal films have been approved in the three major regions of the world including US, EU and Japan for prescription. It seems that these films will dominate over other oral dosage forms having same active pharmaceutical agents and their market will significantly grow[1]. Buccal adhesive films are new drug delivery system, which are made by using mucoadhesive polymer. They have been recently interested due to avoidance of the first pass effect and ability to sustain release for topical and oral therapy. Buccal film is preferred over adhesive tablets and oral gels—due to flexibility, comfort and the relatively long residence time on the mucosa. Moreover, the buccal film has an added advantaged of -protecting wound surfaces, thus reducing pain as well as treating oral diseases more effectively. The mucoadhesive film can be bi-layer for unidirectional release and prevent absorption from GIT[2]. Bi-layer films can be a carrier of more than one drug at a time and serve as a drug delivery system with potential of providing diversity and adaptations. They may be used to incorporate two drugs separately in individual layers. Moreover, mucoadhesive bi-layer films entails added benefits of being manipulated in a way to develop one layer as IR layer and second as a SR layer. IR layer is used to provide rapid release of the drug [3] and hence, faster onset of action while sustained release ensures controlled and SR of the drugs[4]. TZN is a myotonolytic agent and considered an effective agent for the treatment of spasticity. It has to face the extensive hepatic metabolism when administered through oral route due to which its bioavailability is limited only to 30 to 40%. Its half-life is thus reduced considerably, making it to be administered in repeated doses. All these factors contribute in the non-compliances of the patients. MLX is a non-steroidal anti-inflammatory drug and is effective in the treatment of various pain disorders. It belongs to class II in the biopharmaceutical classification system. Poor water solubility may cause decrease in its bioavailability and hence effectiveness. Buccal films are considered suitable drug delivery system that can be used for rapid absorption of the drug across the buccal mucosa as well as avoidance of first pass metabolism and hence enhancing the bioavailability of the drugs.

The current study was design with the aim of developing such a dosage form, capable of delivering TZN and MLX in combination to provide patient compliance and better relief in pain disorders. It was studied that TZN significantly produced analgesic and anti-inflammatory effects. It not only enhanced the analgesic and anti-inflammatory effect of MLX but also improved its gastrointestinal tolerability[5]. TZN and MLX were loaded in bilayer films and their *in-vitro ex-vivo* and *in-vivo* pharmacokinetics were studied to observe the potential of dosage form to deliver both drugs effectively.

## 2. Materials and methods

### 2.1 Materials

TZN and MLX gifted by Pharmedic Laboratories Lahore Pakistan, Ortho-Phophoric acid (Sigma-Aldrich GmbhChemie, Germany), Diethyl ether (Merck Darmstadt, Germany), Methanol (Merck Darmstadt, Germany), Sodium Hydroxide (Merck Darmstadt, Germany), Dihydrogen potassium phosphate (Merck Darmstadt, Germany), Nitrogen and Double distilled water was taken from the research laboratories of Department of Pharmacy, Bahauddin Zakariya University Multan Pakistan.

All the reagents and chemicals were of HPLC grade.

### 2.2 Methods

#### 2.2.1 Preparation of bilayer buccal films

Initially, SR layer containing TZN and IR layer of MLX were prepared and evaluated according to the method described by M Hanif and M Zaman in 2017, M Zaman, M Hanif and AA Qaiser in 2016[6,7]. Same method was followed for the formulation of bilayer films. Accuracy in the quantity of ingredients was achieved by preparing suitable solutions and dispersions of the materials. ARX was soaked in distilled water to prepare 3% w/v gel, similarly aqueous solutions of glycerol (6%), HPMC K15M (2%), polysuccralose (2%) and TZN (3%) were prepared. Because of hydrophobic nature of MLX, its 3% solution was prepared in methanol containing 0.1 M solution of NaOH (9:1). Required volume of ARX was taken in a beaker and stirred by using hot plate magnetic stirrer (JISICO J-HSD180 Korea). After that glycerol, HPMC K15M, TZN and sweetening agent were added one by one in the beaker under continuous stirring. After confirming complete mixing of all gradients, the mixture was poured in a glass petri dish having a surface area of approximately 24cm^2^ and an inverted funnel was placed over it. Petri dish was placed in hot air oven at 40°C for 24 h to get dried films. 2^nd^ layer of MLX (IR layer) was prepared by pouring solution containing MLX, film former (HPMC E-15), plasticizer (Polyethylene glycol 400), surfactant (Tween 80), sweetener (Polysuccralose/) and flavoring agent (Orange flavor) over the SR layer and get dried as described earlier[8].

#### 2.2.2 *In-vitro* characterizations

Bilayer buccal films were evaluated for various *in-vitro* characteristics such as thickness, weight variation, folding fortitude, surface morphology, surface pH, drug release, drug contents and percentage moisture contents.

#### 2.2.3 *Ex-vivo* characterization

*Ex-vivo* permeation across buccal mucosa by using Franz diffusion cell and *ex-vivo* mucoadhesion strength by using modified physical balance was evaluated.

#### 2.2.4 *In-vivo* pharmacokinetics

Bilayer films were evaluated for their *in-vitro* and *ex-*vivo properties. Each bilayer film was loaded with 3mg, each of TZN and MLX in their respective layers. Various pharmacokinetic parameters were studied using non-compartmental approach and calculated by using PK Solver®. (Microsoft® Excel software 2016).

Experimental animals: Albino rabbits having average weight of 3kg were taken from the animal house, The University of Lahore, Lahore-Pakistan and used for in-vivo pharmacokinetics of TZN and MLX containing buccal films. Rabbits were kept fastened for 24 h prior to the study and allowed access to water ad libitum[9] and study was performed according to the guideline of the local animal ethical committee of Bahauddin Zakariya University Multan and The University of Lahore. The “Instructional animal research ethic committee” of the Faculty of Pharmacy, The University of Lahore, Lahore void ref no “IAEC-2016-18”, approved all animal experiments. After experimentation, the treated animals were kept in continuous care until they regained their health. After providing washout period, they were gifted generously to the animal house, The University of Lahore, Lahore-Pakistan.

Inclusion criteria: Inclusion criteria for experimental animal included; Rabbits with good health; All the rabbits were given no treatment since last two weeks; Rabbits having average weight of 3kg; Male albino rabbits.

Exclusion criteria: Exclusion criteria for experimental animals were; Rabbits showing any sign or symptoms of ailments; Rabbits received treatment for any disease or experimental study; Rabbits having average weight greater or less than 3kg; Female rabbits were excluded from studies.

All rabbits received single dose of TZN (1mg/kg) and MLX (1mg/kg) in the form of buccal films and standard drug solutions/dispersions of both drugs (1mg/kg) (by test and control groups respectively). For administration of mucoadhesive buccal films, the rabbits were slightly anesthetized [10] with an intramuscular injection (i.m) injection of a mixture of diazepam (1mg/kg) and ketamine (10mg/kg). Once the rabbits shown symptoms of anesthesia, a catheter was placed in the marginal ear vein [11]. Buccal films were placed carefully in the buccal cavity and gently pressed to get stuck with mucosal membrane.

Study Design: Total 18 rabbits were used for pharmacokinetic studies. Animals were divided into three groups with total of 6 rabbits in each group (n = 6). Latin square crossover study design was applied in such a way to all the groups that rabbits no 1 and 4 were given buccal film, no 3 and 5 were treated with oral solution, which was considered as standard. After first washout period of 14 days the arrangement of rabbits was such as rabbits 2 and 4 have used oral solution and rabbits 3 and 6 of each group were treated with buccal film. In the 3^rd^ and last period, the selection of dosage forms in albino rabbits were such as rabbits 1 and 6 each group were given oral solution, and rabbits 2 and 5 used buccal film[12].

Sample Collection: Blood samples (1ml of each) from marginal ear vein of the rabbits were collected after several intervals (0.5, 1, 2, 4, 6, 8, 12 and 24h) into heparinized tubes [13].

Extraction of the drugs from rabbit’s plasma: Blood samples were allowed to centrifugation promptly for 10 min at 5000 rpm. The supernatant was separated and subjected to vortex for 10 min after adding 3ml methanol and again centrifuged for 20 min at 5000 rpm. Later, the supernatant was transferred to another test tube along with 3 ml of diethyl ether followed by centrifugation for 10 min at 5000 rpm. After that, the sample was dried at 40°C by using water bath. The dried sample was than reconstituted with 200μl of the mobile phase and out of it, 20μl was injected to the HPLC for analysis.

Area under the curve (AUC): When performing non-compartmental analysis, the area under the concentration-time curve AUC_0-∞_ is calculated to determine the total drug exposure over a period of time together with C_max_, for comparison purposes. The trapezoidal rule was used for the determination of area under the plasma concentration-time curve from 0 to 24 (AUC_0-24_) and from 0 to infinity (AUC_0-∞_ ng h/ml). The latter can be determined from AUC_0-24_ as; AUC_0-∞_ = AUC_0–24_+C*/k (C* is the last measured concentration and k is the elimination rate constant).

$$AUC=\frac{1}{2}(C_{1}+C_{2})(t_{2}-t_{2})$$ (1)

Where t_1_ is the initial time, t_2_ is the final time, C_1_ is the initial concentration and C_2_ is the final concentration of the drug.

Peak plasma concentration (C_max_): It is a term used in pharmacokinetics refers to the maximum (or peak) serum concentration that a drug achieves in a specified compartment or test area of the body after the drug has been administrated and prior to the administration of a second dose. Although C_max_ was quantified through visual inspection of the plasma time-concentration curves but can also be calculated as;

$$C_{max}=\frac{FX_{0}}{V_{d}}\times {exp}^{-ke*}t_{max}$$ (2)

Where Ke is the elimination rate constant, F is the fraction of dose and Vd is the volume of distribution.

Peak time (t_max_): t_max_ is the term used to describe the time at which the C_max_ is observed. t_max_ trough visual inspection of the plasma time-concentration curves but, equation below can also be used for its calculations.

$$t_{max}=2.303\;log\;K_{a}/K_{e})\;(K_{a}-K_{e})$$ (3)

Where Ka is the rate constant and Ke is elimination rate constant.

Plasma half-life (t_1/2_): It is the time when half of the drug is excreted from the body and it can be calculated by the equation;

$$t_{1/2}=0.693/k$$ (4)

Where k is the rate constant.

#### 2.2.5 Statistical analysis

Statistical analysis of the data obtained from the experimental work was performed by using One-way ANOVA with 95% confidence interval followed by Bonferroni's Multiple Comparison Test. GraphPad Prism version 7 was used to conduct this analysis. Mean differences were taken as statistically significant at the level of *p<*0.05. All the trials were performed at n = 6 and data were recorded as mean ± SD.

## 3. Results and discussions

**Fig 1:**
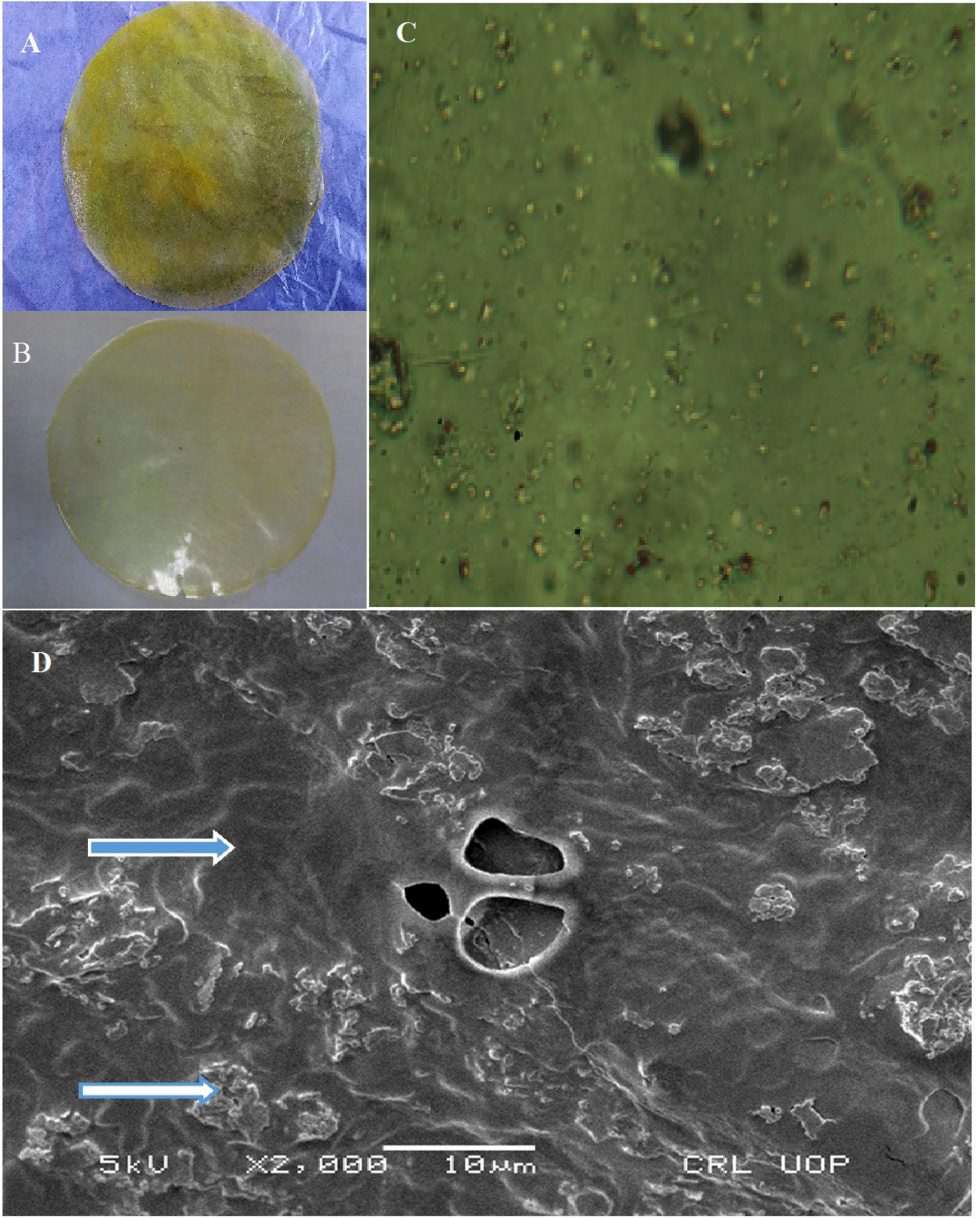
Image taken by the digital camera (Nikon Coolpix p500) showing shiny and smooth surface of the SR layer of TZN (A) and IR layer of MLX (B), Optical microscopic image of bilayer buccal film taken by an optical microscope at 40X (C) and SEM image of bilayer buccal film taken at 2000X (D) showing uniform mixing of ingredients (blue arrow with white outline is indicating polymeric meshwork while white arrow with blue outline is pointing out the imbedded drug).

After performing various trials, a formulation having polymer and plasticizer ratios 3.3:1.0 for IR layer and 4.0:1.0 for SR layer were selected and converted into bilayer films. Results of studied parameters were the indication, that films were of uniform thickness with standard deviation ranged from.±0.21 to.±0.83. Fluctuations in the films’ weight were also in the acceptable limits and surface pH of films was agreeable to that of the buccal cavity, making them suitable for buccal administration. Drug contents were almost touching the hundred marks, designating ARX as polymer having good drug loading capacity. The value of folding fortitude was greater than 300 indicating good mechanical strength of the films. Fabricated films were subjected to mucoadhesion strength and a significant mucoadhesive strength was noticed (Table 1). Surface of the film was found uniform with uniformly distributed drug and polymers (Fig 1).

**Table 1:** Results of studied parameters of buccal films.

Characteristics	Results
Average thickness (mm)	0.282.±0.21
Average weight (mg)	145.78±3.71
Average pH	6.5±0.13
Average moisture contents (%)	5.7±2.3
Folding fortitude	300<
Mucoadhesion strength (N)	1.63 ± 0.21
Drug contents (%)	99.32 (TZN) and 98.72 (MLX)

### 3.1 *In-vitro* release and *ex-vivo* permeation of MLX and TZN

*In-vitro* drug release and *ex-vivo* permeation studies revealed suitable proportion of released and permeated amount of both the drugs. Drug release depends upon the penetration of dissolution medium into the polymeric matrix. The dissolution medium hydrates the swell-able polymer, which swells forming a gel like configuration, dissolves the drug, relaxes the chain of the polymer and resultantly the drug diffuses out. TZN being water soluble, over a wide range of pH is thought to be a good model drug for evaluation of a newer sustained release polymer. The release of TZN from buccal film was retarded efficiently by ARX and it has allowed 87.86% of the drug to release in 8 hrs. It was in-line with the previous studies that ARX has the ability to provide sustained effect[14,15]. PEG 400, which was used as plasticizer in IR layer, is an effective co solvent and can enhance the solubility as well as dissolution rate of the poorly water soluble drugs like MLX. Enhanced dissolution rate and solubility of MLX may be the cause of greater drug release from the formulations **[16]**. IR layer has release about 101.31% of the drug during *in-vitro* dissolution studies.

**Fig 2:**
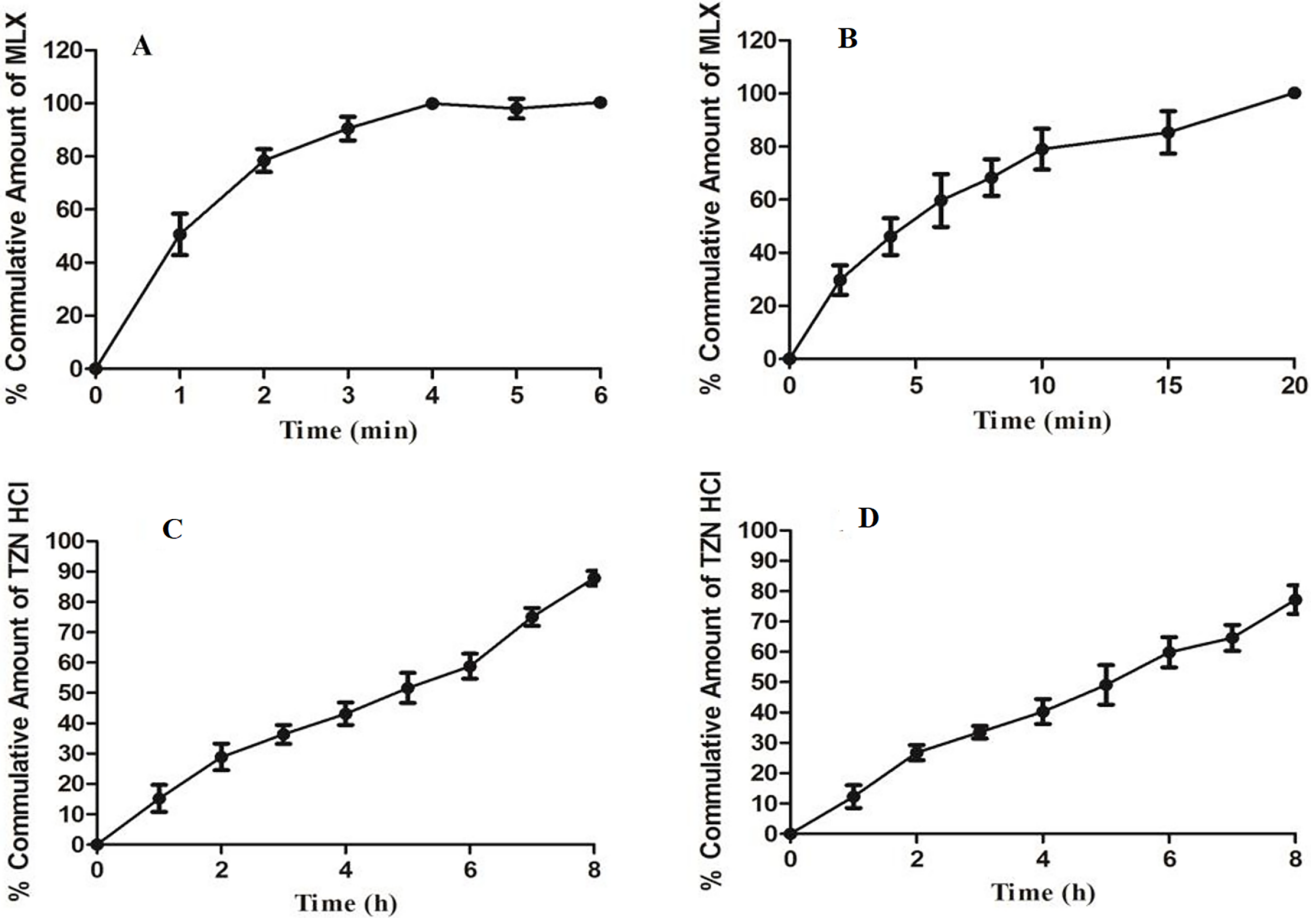
Graphical illustration of *in-vitro* dissolution and *ex-vivo* permeation of MLX and TZN. A and B are showing drug release and permeation of MLX while C and D are describing release and permeation of TZN from bilayer mucoadhesive buccal film.

Drug permeation across mucosal membrane can be a restraining feature for several drugs given through the buccal route. Drug permeation enhancers are proficient of reducing permeation hurdle of the mucosa membrane. A reversible method of reducing the barrier potential of mucosal tissues may be applied to deliver a large number of drugs through this route. The permeation enhancing agent can change the potential of this obstacle safely, causing an increase in drug permeation. Different mechanisms can be adopted by the permeation enhancer to improve the transport of drug across the mucosal membrane and these mechanisms may include; increasing cell membrane fluidity, extracting intercellular lipids, interacting with epithelial protein domains, altering mucus structure and rheology [17]. Plasticizers such as glycerol and PEG 400 can enhance the infiltration of drug across the skin as well as mucosal membranes [18]. Both IR as well as SR have shown good permeation characteristics by allowing 97.32 and 76.89% of MLX and TZN to pass through buccal mucosa during *ex-vivo* permeation studies respectively (Fig 2).

### 3.2 Evaluation of release and permeation patterns of the drugs

Mechanism and pattern of release and permeation have been assessed when the data was subjected to kinetic models. First order kinetics was found to be dominating the release and permeation of MLX from the IR layers of the prepared films. This behavior recommended the dependency of release and permeation over concentration of MLX in the reservoirs. Higher values of Hixson Crowell cube root model proposed uniform exhaustion of the films over the entire duration of the studies. Suitable values of Higuchi model suggested diffusion type of drug release and these recommendations were further strengthen by the goodness of fitness to the Korsmeyer-Peppas model. Values of n<0.5 were the indication that release and permeation have been following fickian type of diffusion (Table 2).

**Table 2:** Kinetics modeling for data of drug release and drug permeation from TZN and MLX containing bi-layer mucoadhesive buccal films.

Kinetic Models		*In-vitro* Dissolution Studies		*Ex-vivo* Permeation studies	
		MLX	TZN	MLX	TZN
**Zero order**	R^2^	0.7191	0.9788	0.8535	0.9813
	k°	5.092	10.756	4.378	9.816
**First order**	R^2^	0.9855	0.9482	0.9937	0.9801
	K^1^	0.251	0.169	0.150	0.148
**Hixson- Crowell**	R^2^	0.9668	0.9829	0.9896	0.9941
	k_HC_	0.052	0.049	0.042	0.043
**Higuchi model**	R^2^	0.8828	0.9618	0.9685	0.9728
	k_H_	23.081	25.335	20.684	23.186
**Korsmeyer- peppas model**	R^2^	0.9613	0.9846	0.9841	0.9944
	k_KP_	43.375	13.323	29.814	13.256
	n	0.265	0.881	0.365	0.833
**Best fit model**		**First order**	**Korsmeyer- peppas**	**First order**	**Korsmeyer- peppas**

k°, k^1^, k_HC,_ k_H_ and k_KP_ are the rate constants for respective kinetic models

Stated mathematical models also applied to study the release and permeation of TZN. The best-fit model was Korsmeyer-Peppas model as it shown the highest values of correlation coefficient (R^2^ = 0.9729 to 0.9944). Buccal film formulation showed non-fickian type of drug release and permeation (n>0.5). Zero order kinetics illustrated good controlled behavior of drug release and permeation as the values of R^2^ were towards higher side (0.9788 to 0.9922). Values of R^2^ greater than 0.9 for Higuchi model and Hixson Crowell cube root suggested that TZN was diffused out uniformly from the entire surface of the films throughout the study period (Table 2).

### 3.3 Drug-excipients compatibility

IR spectra of MLX has explored all the characteristic peaks as its principle absorption peak appeared at 3291, implying the stretching of secondary amine (N-H). Scissoring vibration of NH_2_ was observed at 1620 and C = N stretch at 1540. C-H wagging was seen at 1261.7 and at 1161, S = O stretching has been appeared. Peak at 710 described the rocking of C-H group. The principal absorption peak of HPMC was observed at 3449 cm^-1^, indicating the presence of OH-stretching. At 2898 cm^-1^ CH-stretching and at 1189 cm^-1^ C-O-C-stretching[19] have been noticed. Almost similar and comparable peaks with minor fluctuation were noticed when spectrum of pure drug, HPMC and formulated film were compared. In case of ARX, a broad absorption band was noticed at 3321 cm^−1^, which can be accredited to–OH stretching of alcohols. A peak came into sight at 2931 cm^−1^ that was due to–CH stretching of alkanes. The peak at 1030 cm^−1^ was recognized as C–O–C stretch of ether. The other peaks observed at 895, 717, and 609 cm^−1^ were due to polymer backbone bending and these results were compared to those reported by S. Iqbal, S Saghir and a few other researchers, who have done their work on ARX and find in acquiescence to them[14,20–22].

**Fig 3:**
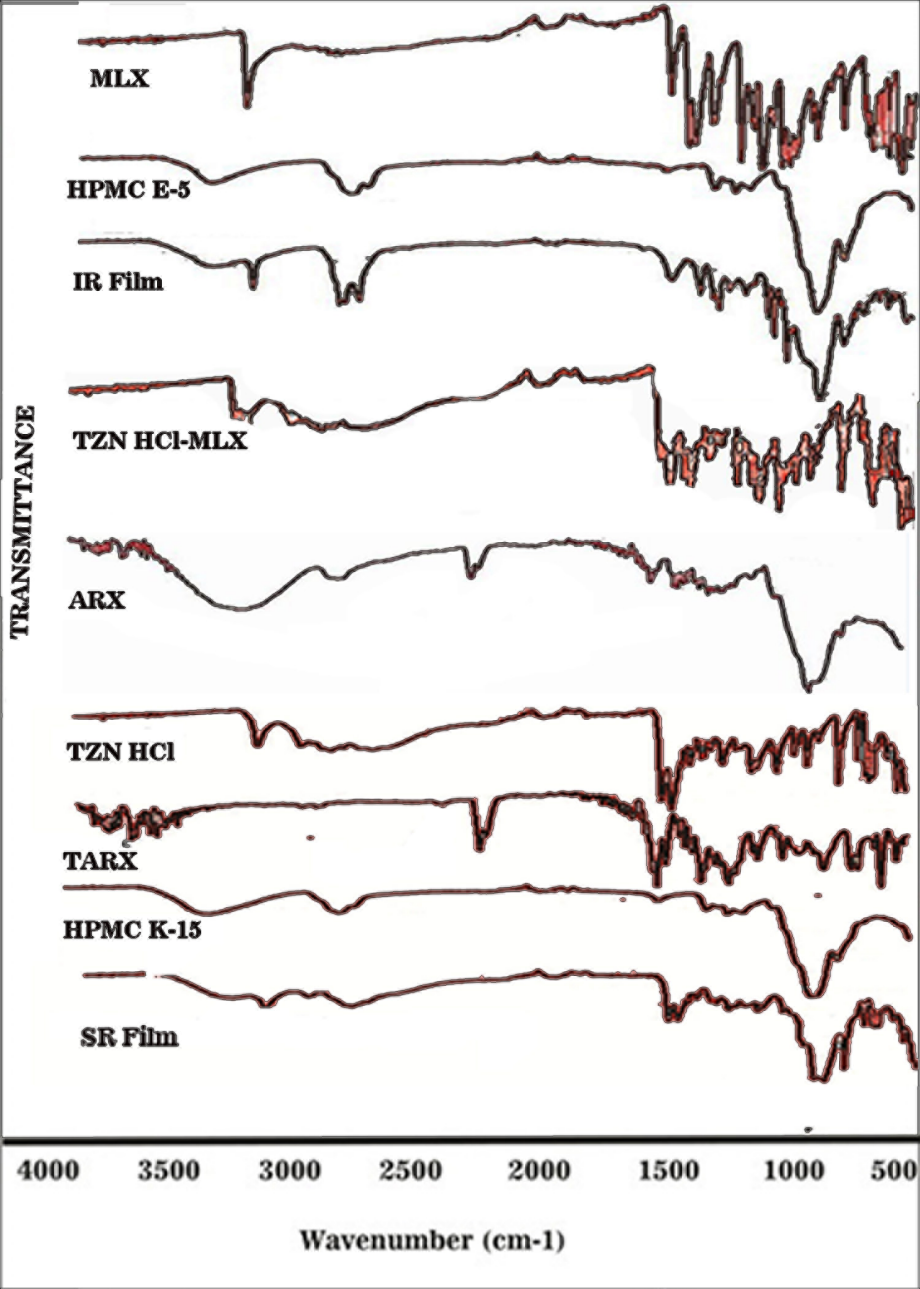
Figure showing FTIR spectrum of different ingredients and prepared films.

FTIR spectrum of formulated mucoadhesive film showed all characteristic peaks with negligible variation or shift in the characteristic peaks of the ingredients, suggesting that the selected combination of the ingredients is suitable and has no chemical interaction between each other (Fig 3).

### 3.4 *In-vivo* pharmacokinetics

**Fig 4:**
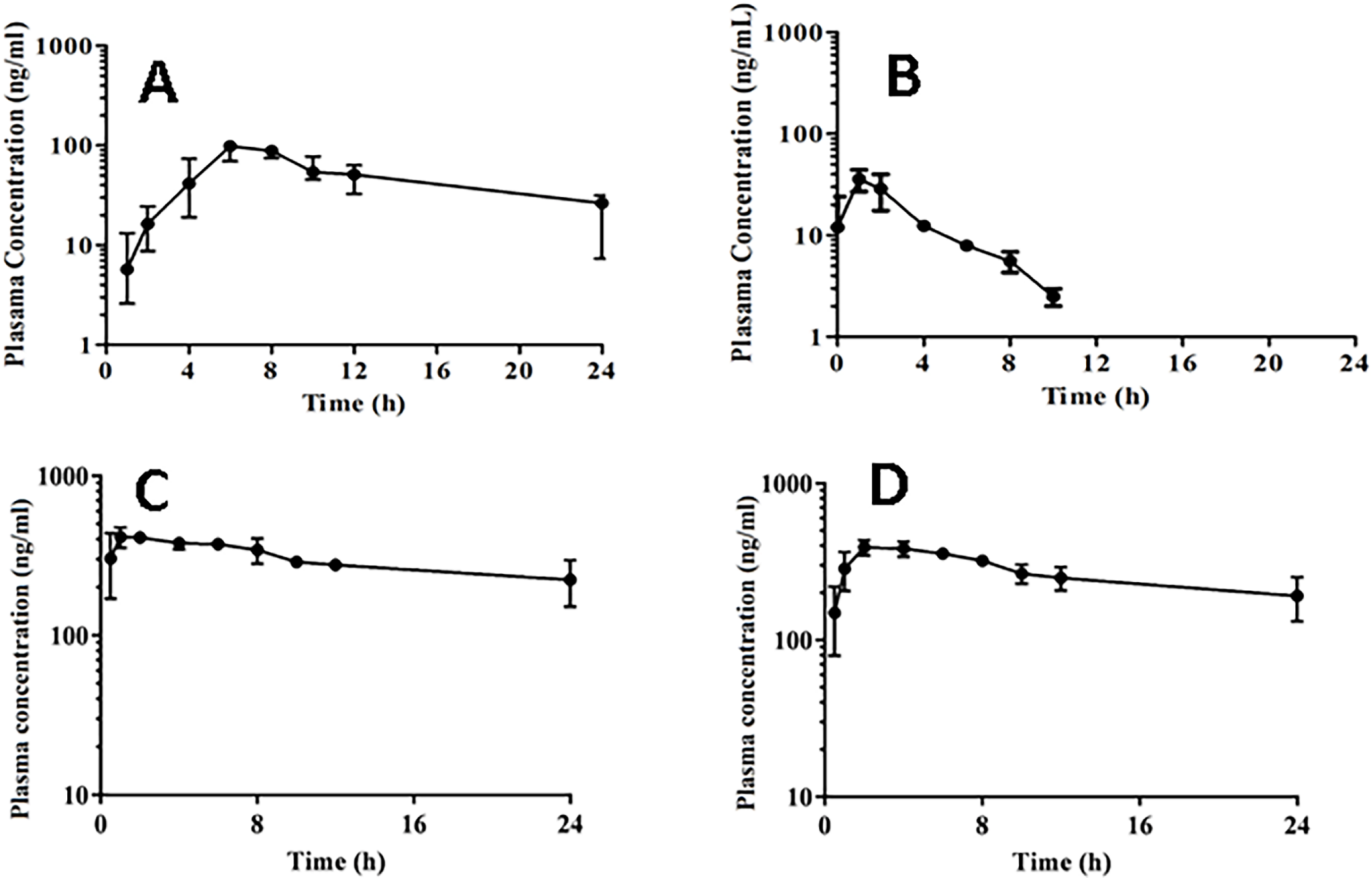
Pharmacokinetics of TZN and MLX after administration of SR films and standard oral solution containing 1mg/kg of the drug. Absorption of TZN from buccal films (A) and from oral solution (B), and MLX from buccal film (C) and oral dispersion (D).

The liquid chromatographic technique (HPLC) was used to estimate the pharmacokinetics of TZN and MLX in rabbit’s plasma as well as to inspect the *in-vivo* performance of buccal film. Oral solutions of TZN and dispersion of MLX were used as standard in a crossover study design applied on albino rabbits. Detailed of inclusion and exclusion criteria has already described in the materials and method session. The plasma concentration-time profiles of both drugs following absorption across the buccal mucosa were exemplified in Fig 4 and the results of concerning pharmacokinetic parameters have been outlined in Table 3.

**Table 3:** Pharmacokinetics of TZN and MLX after administration of buccal films and standard oral solution/dispersion containing 1mg/kg of the drugs.

Parameters	TZN		MLX	
	Buccal Film	Oral Solution	Buccal Film	Oral Dispersion
*t*_1/2_ (h)	9.77	2.51	19.1	20.5
*t*_max_ (h)	6	2	2	2
C_max_ (ng/ml)	93.55	42.18	447.9	411.33
AUC _0-t_ (ng/ml*h)	1043.4	149.1	6773.66	6475.43

PK Solver has been used for calculation of results, which were analyzed by One-way ANOVA followed by Bonferroni's Multiple Comparison Test using Graph Pad Prism version 7.0. Results of pharmacokinetic parameters following administration of TZN containing buccal film formulation showed that values of C_max_ were 91.92ng/ml, 94.62ng/ml and 94.12 ng/ml in groups A, B and C respectively. Half-life (*t*_*½*_) was 9.91h, 9.49 and 9.92h while a constant *t*_max_ (6h) was achieved in all three groups. The results were compared to that of standard oral solution, which showed average C_max_ 42.2 ng/ml, 39.98 ng/ml and 44.38 ng/ml in A, B and C respectively and the average *t*_*½*_ about 2.5hr. There is an increment of more than 2 fold in bioavailability and greater than 3 fold in half-life. These results were the evident of the remarkable increase in the plasma concentration of TZN after following buccal administration of the films. The results obtained after the administration of oral solution in the current study and previously reported by Henney, H.R *et al*. 2008 after oral administration of TZN were comparatively less than those obtained after administration of buccal film[23]. This pointed to an improved pharmacokinetic profile of the drug in terms of buccal films. However, results of *t*_max_ were agreeable to those studied by Galal M. and his companion researchers in 2014[24]. The results for AUC_0-24_ and AUC_0-∞_ were 1043.438 and 1354.777 ng h/ml respectively in-group A, 1120.365 and 1479.557 ng h/ml in group B and 1099.76 ng h/ml and 1434.69ng h/ml in group C respectively. Higher values of stated parameters as compared to the results of standard solution of TZN were the clue of greater drug release and drug absorption in the blood circulation. Additionally, the obtained results were comparatively better than those calculated by Fatima A and research group in their studies[25].

As illustrated in Fig 4, MLX was instantly absorbed and reached maximal concentration at 1h in all three groups of rabbits and these results were incompliance to those obtained by Montoya L and his co-workers in 2004[26]. Average C_max_ of MLX achieved in the study was comparatively better that reported by Patricia V Turner and B. Kimble in their studies[27,28]. MLX from standard oral dispersion has taken a bit more time to reach C_max_. These results had shown that there was rapid release of drug from IR film that might be the cause of ultimate improvement in the bioavailability of drug. This rapid release of drug might be due to larger surface area, less thickness of the film and greater solubility of the film in the presence of tween 80 and PEG 400. Both Tween 80 and PEG 400 are known to enhance the dissolution and solubility of the less soluble drugs[16,29]. The average *t*_*1/2*_ of MLX was 19.1 hr and results were comparable to those obtained by Patel and Amin in 2011, and Amanda J Kreuder and his co-worker in 2012[30,31] but smaller for MLX from oral dispersion (20.5h). Smaller *t*_*1/2*_ and greater C_max_ (447.9 ng/ml) were the indication of better dissolution profile and rapid bioavailability of drug from prepared formulation. Hence, it is proved that selected combination of the ingredients was suitable for the formulation of MLX as IR films. The average values of AUC_0-24_ and AUC_0-∞_ for MLX obtained from buccal films were 6773.66 and 11801.25 ng h/ml respectively. The results were better than those obtained from standard oral dispersion of MLX (6475.43 AUC_0-24_ and 10823.8 ng h/ml). However, the results were comparable to the study conducted by Eroglu H and his research group in 2009, Patricia V Turner and B. Kimble in their studies conducted in 2006 and Montoya L and his co-worker in 2004 [27,32].

### 3.5 Statistical analysis

Statistical analysis was performed for the comparative evaluation between formulated films and standard oral solution. TZN, released from buccal film has shown comparatively better pharmacokinetic profile when its results were compared with the outcomes of oral solution (*p<*0.05). It is the clear indication of comprehensive improvement in the pharmacokinetic profile of the drug, administered through buccal rout. MLX delivered through buccal route showed statistically significant outcomes in terms of Cmax and *t*_1/2_ (*p<0*.*05*), Although there was a dissimilarity between the AUC of MLX administered as oral dispersion and buccal films but statistically there was no significant difference (Table 4).

**Table 4:** Statistical data table for pharmacokinetic parameters of TZN and MLX obtained from buccal film and oral solution/dispersion.

Bonferroni's Multiple Comparison Test	Studied Parameters	Mean Difference	T	Significant	*p-*value	*R*^*2*^
TZN films vs Oral Solution	C_max_	44.54	5.579	Yes^b^	0.0009	0.9393
MLX vs Oral Dispersion	C_max_	38.83	3.899	Yes^a^	0.0141	0.7584
TZN films vs Oral Solution	*t*_*1/2*_	8.247	82.22	Yes^c^	0.0001	0.9993
MLX vs Oral Dispersion	*t*_*1/2*_	-1.527	4.046	Yes^a^	0.0192	7324
TZN films vs Oral Solution	AUC	837.5	109.2	Yes^c^	0.0009	0.9996
MLX vs Oral Dispersion	AUC	28.49	2.864	No	0.1138	0.5154
TZN films vs Oral Solution	*t*_*max*_	3.967	121.9	Yes^b^	0.0001	0.9997
MLX vs Oral Dispersion	*t*_*max*_	0.0006	0.1369	No	0.9784	0.0072

^“a”^ is indicating less significance

^“b”^ greater significance and

^“c”^ indicating most significance

“*” is indicating less significance

“**” greater significance and

“****” indicating most significance

## Conclusion

The objectives of the study have productively been achieved as ARX was invariably extracted and subsequently proved to be a key potential SR mucoadhesive film former. It has shown reliable compatibility with TZN as well as capacity of loading sufficient amount of the drug. HPMC is a well known polymer used for pharmaceutical dosage forms and in this project it has proved its ability to deliver MLX, loaded in IR films, promptly. Preparation of IR and SR films evidenced successful not only when they were prepared individually but also when made combine to form single bilayer film. The astounding results demonstrated that the combination of ARX and HPMC proved to be significantly suitable for the preparation of bilayer buccal films to deliver 2 APIs in 2 different release pattern; one rapidly (MLX) and other (TZN) in controlled and sustained manner. They have displayed their effectiveness greatly in two key ways; firstly, by enhancing the patient compliance as a result of reduced number of unit doses of TZN because it increases plasma concentration and half-life; and secondly, by reducing the cost of the medication as a result of merging two drug in single bilayer film. Findings of *in-vitro*, *ex-vivo* and *in-vivo* (pharmacokinetics using HPLC) studies conducted in this research significantly support their use as a carrier of MLX and TZN in treating various pain conditions where combination therapy of NSAIDs and muscle relaxants is recommended.
